# Construction and evaluation of two computational models for predicting the incidence of influenza in Nagasaki Prefecture, Japan

**DOI:** 10.1038/s41598-017-07475-3

**Published:** 2017-08-03

**Authors:** Fei He, Zhi-jian Hu, Wen-chang Zhang, Lin Cai, Guo-xi Cai, Kiyoshi Aoyagi

**Affiliations:** 1 Department of Epidemiology and Health Statistics, School of Public Health, Fujian Medical University, Fuzhou, Fujian 350108 China; 2 Fujian Province Key Laboratory of Environment and Health, School of Public Health, Fujian Medical University, Fuzhou, Fujian 350108 China; 3 Department of Preventive medicine, School of Public Health, Fujian Medical University, Fuzhou, Fujian 350108 China; 4 Institute of Tropical Medicine, Nagasaki University, Nagasaki, 852-8523 Japan; 5 Nagasaki Prefectural Institute of Environmental Research and Public Health, Nagasaki, 2-1306-11 Japan; 6 Department of Public Health, Nagasaki University Graduate School of Biomedical Sciences, Nagasaki, 852-8523 Japan

## Abstract

It remains challenging to forecast local, seasonal outbreaks of influenza. The goal of this study was to construct a computational model for predicting influenza incidence. We built two computational models including an Autoregressive Distributed Lag (ARDL) model and a hybrid model integrating ARDL with a Generalized Regression Neural Network (GRNN), to assess meteorological factors associated with temporal trends in influenza incidence. The modelling and forecasting performance of these two models were compared using observations collected between 2006 and 2015 in Nagasaki Prefecture, Japan. In both the training and forecasting stages, the hybrid model showed lower error rates, including a lower residual mean square error (RMSE) and mean absolute error (MAE) than the ARDL model. The lag of log-incidence, weekly average barometric pressure, and weekly average of air temperature were 4, 1, and 3, respectively in the ARDL model. The ARDL-GRNN hybrid model can serve as a tool to better understand the characteristics of influenza epidemic, and facilitate their prevention and control.

## Introduction

Influenza virus is the most common cause of acute respiratory illness^1^. Although influenza infection is usually self-limiting, it affects all age groups around the world and cause severe complications in high-risk individuals such as children, the elderly and those with chronic medical conditions. In Japan, influenza was designated in 1947 as a notifiable disease under the Japanese Communicable Disease Prevention Law, and systematic surveillance of influenza/influenza-like illness started in 1981. It has also been designated as a notifiable disease to be reported from sentinels (Category 4b) under the Infectious Disease Control Law in Japan since 1999^2^. To date, the dominant viruses causing influenza endemics in Japan include influenza A subtype H3N2 (since 1977), influenza A subtype H1N1 (since 1988), and influenza B. A prominent example of recent influenza pandemic in Japan is the influenza A (H1) pdm09, which first occurred in 2009 and affected 25 million people. Influenza has continued to impose a significant impact on the Japanese population through the 2010/2011 and 2013/2014 seasons^3^.

The prevalence of seasonal influenza epidemics is associated with many different factors such as virus variation, climate and environmental changes, and public health interventions including vaccination. The transmission pattern of influenza and its seasonal variation have long been reported worldwide^4^. Over the past two decades, efforts have been made to develop mathematic models to analyze the characteristics of influenza epidemics, and, more importantly, to forecast outbreaks. The most widely used models for influenza forecasting include the time series autoregressive integrated moving average (ARIMA) model, generalized linear regression (GLM) model, and Bayesian network. Forecasting the timing, extent and duration of influenza outbreaks will be invaluable in guiding planning and implementing effective intervention measurements. However, accurate forecasting remains highly challenging. The 2009 Google Flu Trends (GFT) article^5^ was highly- publicized. However, in 2014 Lazer *et al*. reported that the number of influenza-like outpatients reported by nation-wide CDC laboratory monitoring in 2013 was twice that predicted by GFT prediction service compared by using CDC, which came to the conclusion from laboratory-based monitoring reports across America, indicating the failure of the GFT was recognized as a defective model^6^, because of big data hubris and change of algorithm dynamics. Key words that employed by GFT model may thought having nothing to do with influenza. For example, when “fever” was searched, the key word as “flu” would be suggested. In addition, recommended search could also add the searched frequency of certain popular words. The relative high-frequency key words would always be used by GFT, therefore search engine algorithms would produce adverse effect on forecast results of GFT.

In this study, we constructed two mathematical models including an auto-regressive distributed lag model (ARDL) and a hybrid model integrating ARDL with a generalized regression neural network (GRNN), designated as ARDL-GRNN. We validated the capacity of these two models to model and forecast influenza incidence, using influenza incidence data collected in Nagasaki prefecture between 2006 and 2015 by the Japanese infectious disease surveillance systems (http://www.nih.go.jp/niid/en/idsc-e.html). The performance of these two models was compared to identify the best influenza forecasting model.

## Results

### General characteristics of influenza incidences in Nagasaki, Japan

**Figure 1:**
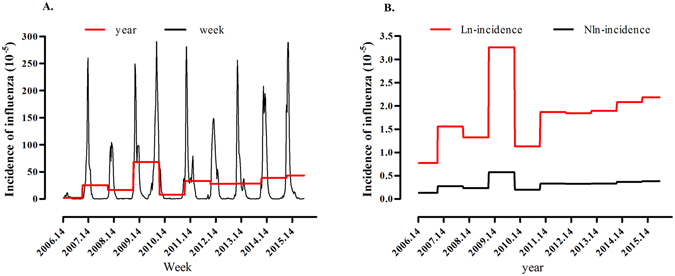
(**A**) The sequence chart of the influenza incidence in Nagasaki prefecture from the 14^th^ week of 2006 to the 36^th^ week of 2015. (Black lines represent the weekly incidence of influenza; Red lines represent the yearly incidence of influenza). (**B**) The sequence chart of the influenza Nln-incidence and influenza ln-incidence in Nagasaki prefecture from the 14^th^ week of 2006 to the 36^th^ week of 2015. (Black lines represent the year Nln-incidence of influenza; Red lines represent the year ln-incidence).

Figure 1A shows weekly incidence of influenza between 2006 and 2015 in Nagasaki, Japan. Influenza occurred in each of the 492 weeks or 123 months surveyed, indicating that influenza infection occurs throughout the year, while incidence almost always peaks between November and January, or at least in winter and early spring. Figure 1B shows the transformed data, Nln-incidence of influenza, in Nagasaki.

### Relationship between influenza Nln-incidence and meteorological factors

To assess the relationship between influenza Nln-incidence and meteorological factors we used the Spearman’s rank correlation test as meteorological factors were non-normally distributed. Nln-incidence of influenza correlated statistically significantly with each of the 13 meteorological factors surveyed except for hours of sunshine per week and weekly average of cloud cover per week (Table 1). In addition, we built a regression model to further analyze the relationship of influenza Nln-incidence with factors, such as weekly average air temperature, precipitation, wind speed, barometric pressure, and relative humidity.

**Table 1:** The relationship between the log-incidence of influenza and the meteorological factors.

Meteorological actors	*M* (*P*_*25*_ *-P*_*75*_)	Spearman’s rank correlation coefficient	*P*
average weekly air temperature	17.18(10.30–23.21)	−0.665	<0.001
average weekly daily maximum air temperature	20.99(13.83–26.18)	−0.652	<0.001
average weekly daily minimum air temperature	13.86(6.93–20.68)	−0.672	<0.001
highest air temperature per week	23.70(17.32–28.45)	−0.642	<0.001
lowest air temperature per week	10.92(3.75–18.75)	−0.670	<0.001
weekly precipitation	22.70(5.97–52.99)	−0.131	0.006
maximum precipitation per week	16.25(4.34–37.77)	−0.128	0.007
duration of sunshine per week	33.54(23.43–44.99)	−0.065	0.175
average weekly wind speed	3.41(2.94–3.84)	0.256	<0.001
maximum wind speed per week	9.62(8.49–10.67)	0.280	<0.001
average weekly barometric pressure	13.73(8.30–23.19)	−0.645	<0.001
average weekly relative humidity	73.40(67.50–80.60)	−0.473	<0.001
weekly average of cloud cover per week	6.80(5.50–8.04)	−0.077	0.109

This regression model was significant (*F* = 178.40, *P* < 0.001). We observed a strong correlation between influenza incidence and weekly average air temperature (*t* = −6.777, *P* < 0.001), weekly average barometric pressure (*t* = 2.015, *P* = 0.044). These observations indicate that an increase in air temperature of 1 °C was associated with a reduction in influenza incidence of 0.0413 units; an increase of 1 Pa in weekly average barometric pressure was associated with an 0.0109 unit increase in incidence. Therefore, we constructed the ARDL model using weekly average air temperature and weekly average barometric pressure.

### The best-fitting ARDL model

First, we performed the augmented Dickey–Fuller (ADF) test according to SC criterion with unit root in level data and included an intercept in the equation. We found that the time series data of the Nln-incidence of influenza (*t* = −6.313, *P* = 0.010), weekly average air temperature (*t* = −7.596, *P* = 0.010), and weekly average barometric pressure (*t* = −7.572, *P* = 0.010) were all stationary series.

Then we chose to construct an ARDL model to analyze these time series data. Using the bounds testing approach of cointegration relationships, the *F*-statistic was 15.532, which far exceeded even the 1% critical value for the upper bound (I_0_ = 5.15, I_1_ = 6.36). Accordingly, we assumed a long-run relationship between variables.

**Figure 2:**
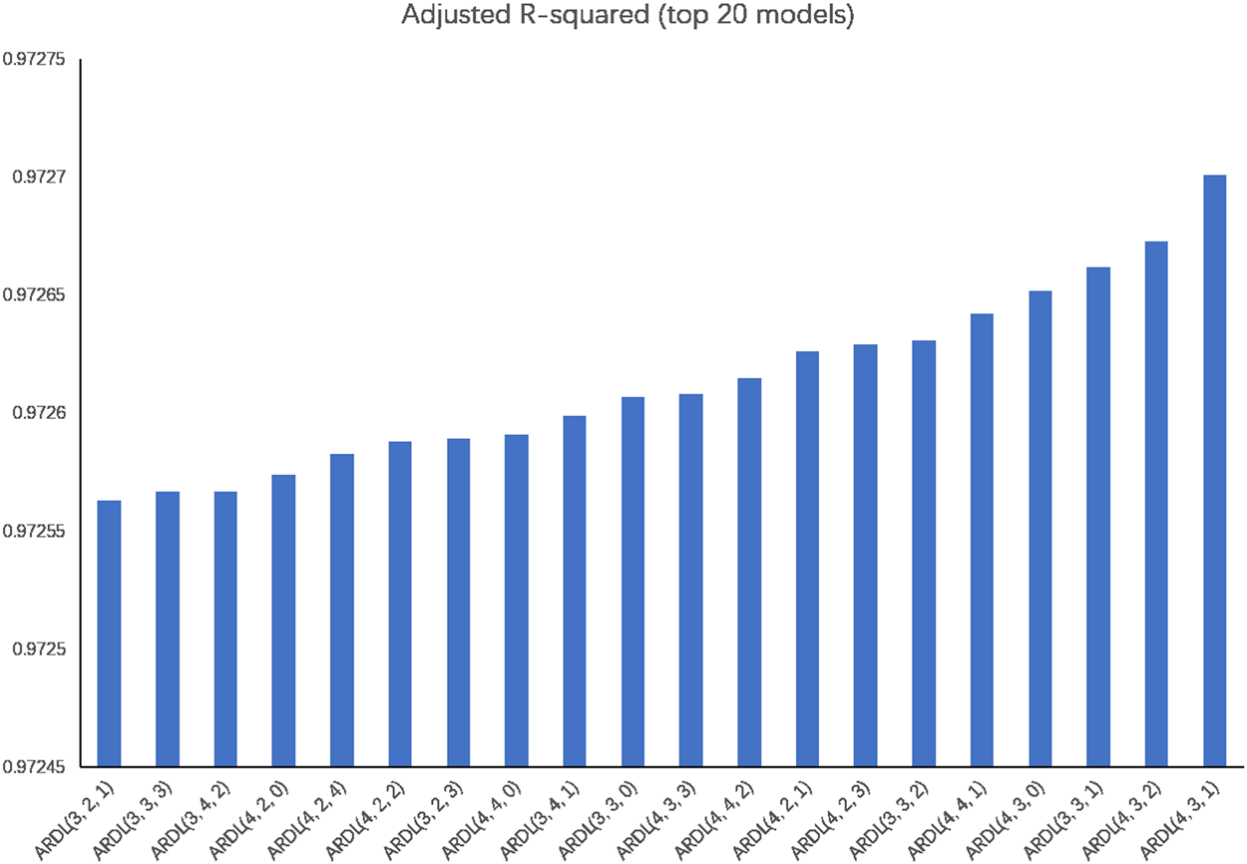
The top 20 ARDL ordered by adjusted R^2^. The ARDL model lag of Nln-incidence of influenza was four, the lag of weekly average barometric pressure was one and the lag of weekly average air temperature was three. The adjusted R^2^ value was chosen to determine the optimal lag order of the variables in ARDL model.

Before the model was built, the key parameter of lag order should be confirmed. According to the criterion of adjusted R^2^ we set four as the maximum dependent lags and determined the lag order for each series variable after evaluating 100 models (Supplementary Table 1). In total, 100 ARDL models were evaluated, the ARDL (4,1,3) model remained. While 20 models performed well, the ARDL model (4,1,3) (Table 2) in which the lag of Nln-incidence was four, the lag of weekly average barometric pressure was one, and the lag of weekly average air temperature was three, was selected for further analyses (Fig. 2). Based on the ARDL (4,1,3) model, the weekly average air temperature (*P* < 0.001) and weekly average barometric pressure (*P* = 0.0027) were significantly associated with the incidence of influenza (Table 3). The formula for the error-correction model was expressed as: the Nln-incidence of influenza − (0.0735 × weekly average barometric pressure + 0.1172 × weekly average air temperature + 1.1271) (*F* = 1547.405, adjusted *R* ^*2*^ = 0.9727, AIC = −3.015 and SC = −2.912) (Table 3). We found that that the current Nln-incidence of influenza was significantly associated with the Nln-incidence of influenza four weeks prior, average barometric pressure one weeks prior, and weekly average air temperature three weeks prior.

**Table 2:** The parameters of selected ARDL (4,1,3) model.

Variable	Coefficient	Standard Error	t-Statistic	*P*
Nln-incidence of influenza (−1)	1.267491	0.048202	26.29540	<0.001
Nln-incidence of influenza (−2)	−0.245196	0.077996	−3.143685	0.0018
Nln-incidence of influenza (−3)	−0.033365	0.074708	−0.446602	0.6554
Nln-incidence of influenza (−4)	−0.056554	0.044652	−1.266554	0.2060
barometric pressure	0.002365	0.001961	1.205900	0.2285
barometric pressure (−1)	0.002607	0.001966	1.326359	0.1854
average weekly air temperature	−0.003733	0.002441	−1.528967	0.1270
average weekly air temperature (−1)	−0.011915	0.002535	−4.700947	<0.001
average weekly air temperature (−2)	0.005619	0.001704	3.298203	0.0011
average weekly air temperature (−3)	0.002103	0.001429	1.471199	0.1420
Fixed regressors	0.076219	0.012589	6.054227	0.0000
R-squared	0.973330	Mean dependent var		0.314721
Adjusted R-squared	0.972701	S.D. dependent var		0.320280
S.E. of regression	0.052918	Akaike info criterion		−3.015187
Sum squared resid	1.187330	Schwarz criterion		−2.912132
Log likelihood	666.8032	Hannan-Quinn criter.		−2.974513
F-statistic	1547.405	Durbin-Watson stat		2.017523
Prob (F-statistic)	<0.001

**Table 3:** Long-run coefficient and error-correction of the ARDL model.

Variable	Coefficient	Standard Error	*t*-Statistic	*P*
Long-run coefficients				
average weekly air temperature	−0.117198	0.028129	−4.166445	<0.001
average weekly barometric pressure	0.073526	0.024407	3.012577	0.0027
intercept	1.127094	0.130090	8.663950	<0.001
Error-correction model				
D (the log-incidence of influenza (−1))	0.335115	0.046407	7.221250	< 0.001
D (the log-incidence of influenza (−2))	0.089919	0.047041	1.911494	0.0566
D (the log-incidence of influenza (−3))	0.056554	0.044652	1.266554	0.2060
D (average weekly barometric pressure)	0.002365	0.001961	1.205900	0.2285
D (average weekly air temperature)	−0.003733	0.002441	−1.528967	0.1270
D (average weekly air temperature (−1))	−0.005619	0.001704	−3.298203	0.0011
D (average weekly air temperature (−2))	−0.002103	0.001429	−1.471199	0.1420
ECM (−1)	−0.067624	0.012261	−5.515528	<0.001

### ARDL-GRNN hybrid model

**Figure 3:**
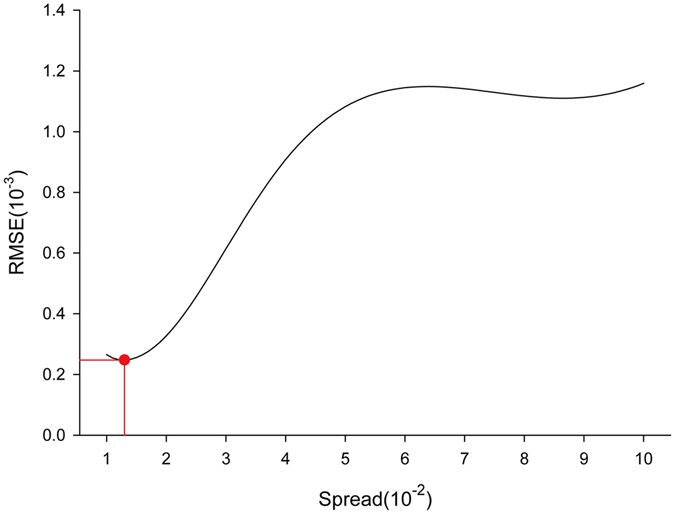
Selection of the optimal spread of the ARDL-GRNN hybrid model in training samples. When the spread is 0.013, the RMSE (the red point) 2.48 × 10^−4^ of the training samples is the least. In principle, if the RMSE for the training samples is the least, the spread is the best.

Because the lag order of Nln-incidence of influenza was four and only the weekly incidence between the 18^th^ week of 2006 and the 35^nd^ week of 2014 were predicted by the ARDL model. The resulting prediction data and their corresponding time values were fed into the GRNN model. To determine the optimal spread factor, the Nln-incidence data of the 1^st^ week of 2008 and the 30^th^ week of 2013 were randomly selected as testing samples. In principle, if the RMSE for the testing samples is the least, the spreading factor will be the best-fitting. To find the minimum RMSE, we set the spread factor between 0.01 and 0.2 with an interval of 0.001 to avoid overfitting. Through trial and error we determined that the spread factor of 0.013 was associated with the lowest RMSE for the testing samples (Fig. 3). Hence, we set up the GRNN model with the RMSE 2.48 × 10^−4^.

To test the forecasting capacities of the ARDL-GRNN hybrid model, the predicted incidence values generated by the ARDL (4,1,3) model for the period from the 36^th^ week of 2014 to the 36^th^ week of 2015 were used as the input. The resulting forecasted incidence values were compared with actual observed data as described below.

### Comparison of modelling and forecasting performance

**Figure 4:**
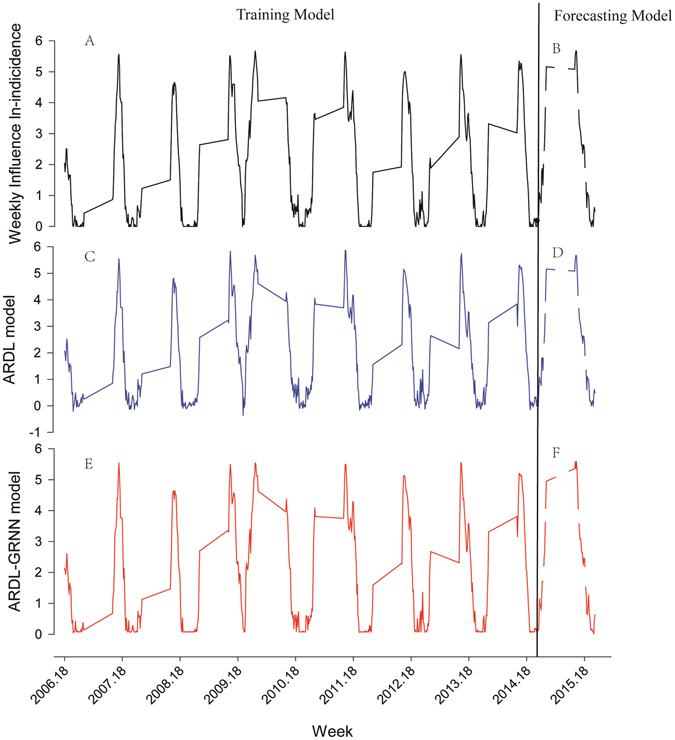
Comparison of actual, predicted and forecasted week incidence of influenza in Nagasaki prefecture, Japan. Training model includes: Actual weekly incidence of influenza by training model (**A**), Best-fitting ARDL training model (**C**), and ARDL-GRNN training model (**E**). Forecasting model includes: Actual weekly incidence of influenza by forecasting model (**B**), Besting-fitting ARDL forecasting model (**D**), and ARDL-GRNN forecasting model (**F**).

Figure 4 illustrates the comparison of the fitting degree and forecasting performance of the ARDL and ARDL-GRNN models. Both models fitted and predicted the observed incidence of influenza well. In both the modelling and forecasting stage, the ARDL-GRNN hybrid model showed lower error rates including the RMSE and MAE compared to the ARDL model (Table 4).

**Table 4:** Comparison of the modeling and forecasting performance of ARDL and ARDL-GRNN models.

Model	Training performance		Forecasting performance	
	RMSE	MAE	RMSE	MAE
ARDL	0.05224	0.03900	0.05124	0.04206
ARDL-GRNN	0.04730	0.03446	0.03453	0.02841

## Discussion

In the current study, we established two computational models, the ARDL and ARDL-GRNN, and evaluated their performance in modelling and forecasting influenza incidence using influenza incidence data and meteorological data in Nagasaki Prefecture, Japan. The ARDL-GRNN hybrid model models and forecasts influenza incidence better than the ARDL model. To the best of our knowledge, this is the first study to integrate ARDL and GRNN to identify the best model for forecasting influenza incidence. The performance of the ARDL-GRNN model suggests that it can serve as a tool to better understand the characteristics of epidemics, and facilitate the prevention and control of influenza.

The most frequently used time series analysis method is the ARIMA model, also known as the Box-Jenkins model^7^, which was originally developed for econometric and environmental time series analyses, and has been expanded to various new models such as seasonal ARIMA and fractional ARIMA. Many infectious diseases, including influenza, exhibit secular trends and seasonal variation due to pathogen strain variation, climate, socioeconomic changes, and health interventions including vaccination. The ARIMA model, including its expanded versions, has become a popular tool in the epidemiological analysis of infectious diseases^8, 9^. Usually, this model is used for fitting without independent variables and relies on retrospective dependent variable data. Additive models are required to study other factors that may affect the incidence or mortality of a specific disease. About 20 studies have been published describing the GLM model or generalized additive models as standard models for analyzing the relationship between influenza epidemics and environmental factors^10^. Also, other time-series data, such as wavelet analysis, has also been used for influenza forecasting^11^.

We speculated that the influence of weather conditions on influenza epidemics involved a temporal lag, so we selected the ARDL model proposed by Pesaran and Shin^12, 13^ as the base for our model. The ARDL model has seldom been used in infectious disease epidemiology. One advantage is suitable for time series data analysis with a small sample size. Additionally, the other advantage of yielding consistent estimates of the long-run coefficients that are asymptotically normal irrespective of whether the underlying regressors are I(1) or I(0).

Secular trends and seasonal variation are crucial contributors to influenza incidence (Fig. 1). We constructed the ARDL model based on the multiple linear regression (MLR) ordinary least-squares (OLS) methods^14^, and found that the current incidence of influenza could be affected by the air temperature three weeks prior and barometric pressure one week prior. Although the incubation time for influenza virus was only two days, this time lag highlighted the delay between virus infection personally and observed incidence shifts of influenza in population. It was reported that the time lag between changes in influenza incidence and changes in temperature and barometric pressure need at least 1–3 weeks^15^. Moreover, as a result of geographical factors, the Mongolia cold high pressure impacts the weather in Nagasaki Prefecture^16^. When the temperature is colder, the air pressure is higher, and spreads more easily^17^.

To overcome the possibility that the ARDL model may not efficiently extract non-linear characteristics of weekly incidence data, we sought to construct a hybrid model by integrating ARDL with GRNN. In contrast to the back-propagation neural network (BPNN)^18^, GRNN includes a special form of the radical basis function neural network in the training stage, which has several advantages over BPNN, including a fast training time, easy parameter settings, and great stability. The spread factor plays an important role in function approximation. On the one hand, a lower spread factor leads to a steeper radial basis function, thus yielding fitting values closer to the actual values, but with poor generalization. On the other hand, a greater spread factor can make the fitting curve smoother, but weakens performance. In the modelling training process, we determined the optimal spread factor following the method of Specht^19^. Since the time series data for influenza incidence in Nagasaki showed a strong seasonal variation and secular trend, we believed it necessary to use the time values as GRNN inputs. Our comparative analysis revealed that the ARDL-GRNN model had a higher prediction accuracy than the ARDL-ECM model that used only the estimated weekly incidence values. Our results agree with published studies in which integration of GRNN into other models such as ARIMA, allowed improved modelling and forecasting accuracy in studies of infectious disease and environmental health^20, 21^.

However, our conclusions are limited by the scope of this study. As recently reported^22^, when analyzing time series data, we are confronted with five issues including changes in immune population, strong autocorrelations, a wide range of plausible lag structure and association patterns, seasonality adjustments, and large over-dispersion. In our study, we focused on resolving the issue of seasonality and trends, and assessed the characteristics of association patterns and lag structure using the ARDL model. Our study involves influenza incidence data collected between 2006 and 2015, which covers the period of a global influenza epidemic (2009). Although the results of model-fitting met our expectations, we do not know the real impact of the 2009 global pandemic on the local epidemic in Nagasaki. We speculate that the accuracy of our model may be increased by excluding 2009 data, but this possibility awaits further investigation. Finally, this study is limited to only one prefecture and a relatively short time-frame. Clearly, further studies will be required to determine whether the ARDL-GRNN hybrid model could be adapted to assess influenza epidemiology in other regions or epidemiology of other infectious diseases.

## Methods

### Study area and data collection

This study focused on the influenza incidence in the Nagasaki Prefecture of Japan. The Prefecture occupies an area of 4095.55 km^2^ and is located on the island of Kyushu, Japan between 128.06°~130.23° East longitude and 31.59°~34.43° North latitude. Nagasaki is surrounded on three sides by the sea with the second longest coastline of any prefecture in Japan (4,203 km). The sea makes up the majority of the prefecture), and Nagasaki also contains many mountains, peninsulas, capes, bays, and bifurcation lakes. Of the 595 islands in the prefecture, 73 are inhabited. Nagasaki has a typical oceanic climate, warm and rainy, with an average annual temperature of 18.0 °C and average annual rainfall of 1,464 mm.

**Figure 5:**
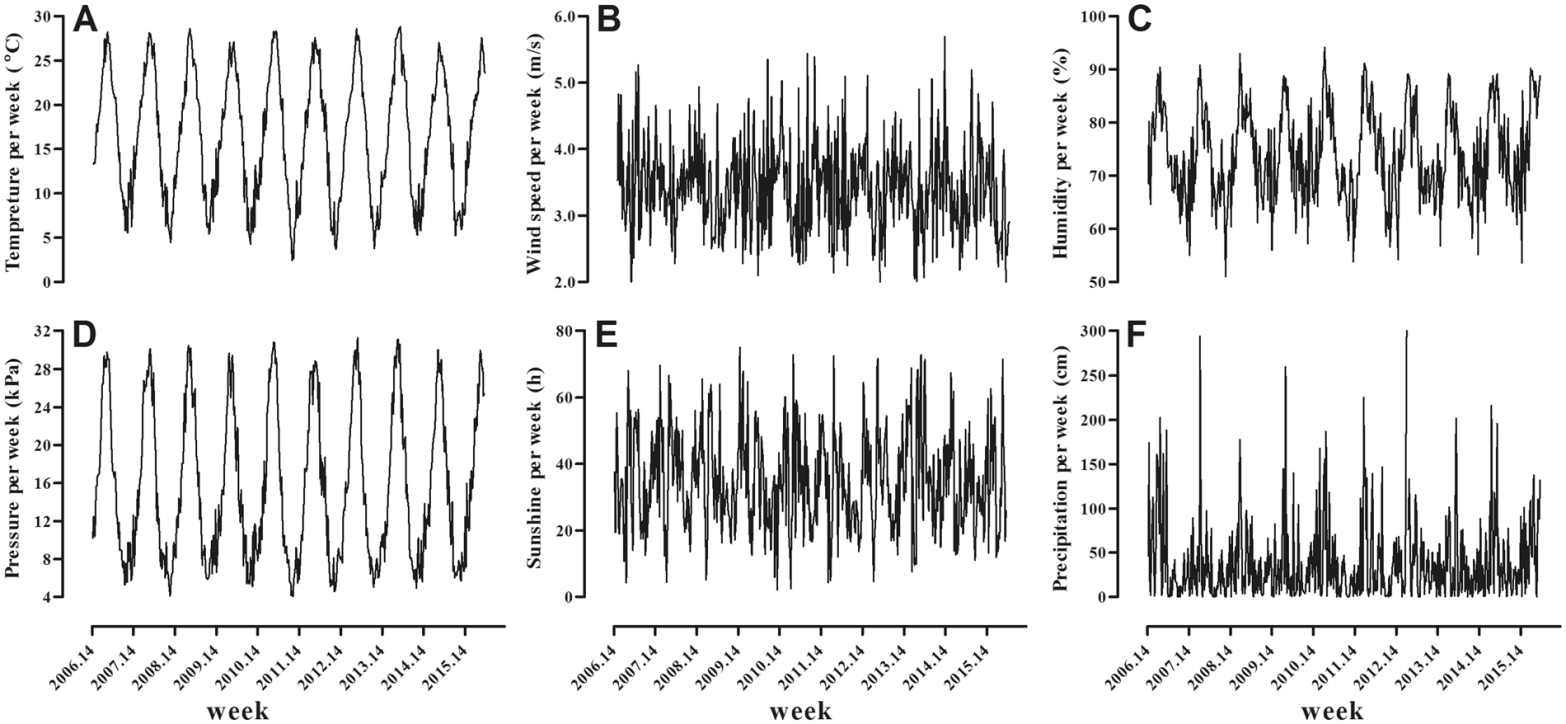
The sequence chart of meteorological data in Nagasaki prefecture from the 14^th^ week of 2006 to the 36^th^ week of 2015. The weekly average air temperature (**A**); wind speed (**B**); relative humidity (**C**); barometric pressure (**D**); and weekly duration of sunshine (**E**); and weekly precipitation (**F**).

The Japanese infectious disease surveillance system collects surveillance data for influenza and 26 other infectious diseases^23^. We retrieved the weekly reported influenza incidence in Nagasaki prefecture between the 14^th^ week of 2006 and the 36^th^ week of 2015. Meteorological data for the same period (Fig. 5) were retrieved from the website of Japan Meteorological Agency (http://www.jma.go.jp).

All the diagnosis of influenza were performed in the hospital, using specific rapid detection kit. However, the diagnosis of type A or B influenza, and virus strain were not detected by the rapid kit. There are 70 stationary monitoring stations in the Nagasaki Prefecture, however, the subtypes of influenza were only monitored by 15 out of all 70 stations. During the non-pandemic period, the subtypes of influenza were only recorded for 15 cases per month (1 case for each station), and during the pandemic period the subtypes of influenza were recorded for 15 cases per week (≥1 cases for each station). Nagasaki Prefecture influenza surveillance data was consistent with that collected elsewhere in Japan. The type and rate of influenza inoculation was also consistent to the country, according to the Japanese health ministry.

### ARDL model construction

The previously described ARDL^12^ model was adapted to predict the influenza incidence and evaluate the long term and short term relationship between incidence rate and different meteorological factors. The training model included 439 weeks of incidence data collected between the 14^th^ week of 2006 and the 36^th^ week of 2014, and forecasting capacity was assessed using 53 weeks of data collected between the 36^th^ week of 2014 and the 36^th^ week of 2015.

The model construction workflow consisted of two steps. The first step was to transform the incidence rate of influenza into ln data to facilitate processing using the Normalization Method. The data was mapped to the 0~1 range using the formula [Normalization ln-incidence (Nln-incidence = ln (incidence-0)/max(incidence-0)]. The correlation between various meteorological factors and the Nln-incidence was assessed using Spearman’s rank correlation and regression analyses. After determining the meteorological factors, the stationarity of the time series was estimated using the ADF Unit Root test.

The second step was to determine the optimal-fitting ARDL and use it to forecast the values of testing time points. First, we used the bounds testing approach to identify any cointegration relationship between the independent variables and the dependent variables in the model, and their direction of action in the presence of cointegration. If the model passed the boundary value test, we estimated the long-term and short-term relationship coefficient. The formula of the ARDL (${\rm{p}},{q}_{1},{q}_{2},\cdots \cdots {q}_{l}$) model was:

1 $$\varphi (L,P){y}_{t}={\sum }_{i=1}^{k}{\beta }_{i}(L,{q}_{i}){x}_{it}+\delta {w}_{t}+{u}_{t}.$$

In which

2 $$\varphi (L,P)={\rm{1}}-{\varphi }_{1}L-{\varphi }_{2}{L}^{{\rm{2}}}-\cdots {\varphi }_{p}{L}^{p}$$

3 $${\beta }_{l}(L,{q}_{i})={\rm{1}}-{\beta }_{{{\rm{i}}}_{{\rm{1}}}}L-{\beta }_{{{\rm{i}}}_{{\rm{1}}}}{L}^{{\rm{2}}}-\cdots {\beta }_{{{\rm{iq}}}_{{\rm{i}}}}{L}^{{{\rm{q}}}_{i}}$$

The long - term dynamic equation was:

4 $$N\,\mathrm{ln}\,-incidenc{e}_{t}=a+\sum _{i=1}^{{n}_{{\rm{1}}}}{\alpha }_{i}{(N\mathrm{ln}-incidence)}_{t-i}+\sum _{i=0}^{{n}_{{\rm{2}}}}{\beta }_{i}M{F}_{t-i}+{\varepsilon }_{{\rm{i}}}.$$

In which n_1_ and n_2_ represent the lag order and MF represent the selected meteorological factors used to build the model. The short-term effect equation was:

5 $$\begin{array}{rcl}{\rm{\Delta }}N\,\mathrm{ln}\,-incidenc{e}_{t} & = & {a}_{0}+\sum _{i=1}^{n}{\alpha }_{1i}{\rm{\Delta }}{(N\mathrm{ln}-incidence)}_{t-i}\\  &  & +\,\sum _{i=0}^{n}{\alpha }_{2i}{\rm{\Delta }}M{F}_{t-i}+EC{M}_{t-1}+{\mu }_{t}\end{array}.$$

ECM is an error correction factor representing hysteresis. The coefficients estimated in Eqs (4) and (5) are significant only in the case of a cointegration relationship. It is necessary to determine the lag order in the model before estimating the long-term coefficients. According to the recommendation of Pesaran *et al*.^13^, we use the adjusted R^2^ criterion to determine the optimal lag order of the variables in the model, taking into account the length of the sample data, and the maximum lag order parameters were set as four using Eviews 9.0 (IHS, Inc. USA). Finally, the best-fitting ARDL was built and applied to forecast the whole complete cycle. The model goodness-of-fit was measured using root means square error (RMSE) and.

### ARDL-GRNN model construction

The ARDL-ECM described above can recognize linear relationships but not non-linear relationships. To overcome this limitation, we integrated the ARDL with the GRNN algorithm. The predicted weekly influenza Nln-incidence values from ARDL and their corresponding time values were fed into the GRNN model. The resulting data were compared with the actual observed weekly incidence values. Since the performance of GRNN depends primarily on the spread factor, we selected the optimal factor after multiple rounds of computation following the method reported by Specht^19^. Two samples randomly selected from the training data set were used as testing samples and all the remaining samples were employed to fit the GRNN model. This ARDL-GRNN hybrid pipeline allowed computation of both linear and non-linear relationships. To compare the prediction accuracy between ARDL and ARDL-GRNN, we estimated the error rates for both models, including the RMSE, mean absolute error (MAE).

All analysis were performed with R 3.3.0 (https://www.r-project.org/) and Eviews 9.0 (IHS, Inc. USA).

### Data availability

Please contact author for data requests.

## Electronic supplementary material

Supplementary Table 1
