# Supplementary material for: Construction and evaluation of two computational models for predicting the incidence of influenza in Nagasaki Prefecture, Japan
Source: Sci Rep. 2017 Aug 3;7:7192. doi: 10.1038/s41598-017-07475-3 (PMC5543162; doi:10.1038/s41598-017-07475-3)
Supplement: Supplementary file 1 — Supplementary Table 1 [file 41598_2017_7475_MOESM1_ESM.doc]

**Construction and evaluation of two computational models for predicting the incidence of influenza in Nagasaki Prefecture, Japan**

Fei He^1^, Zhi-jian Hu^1^*, Wen-chang Zhang^2^, Lin Cai^1^, Guo-xi Cai^3,4^, Kiyoshi Aoyagi^5^

1. Department of Epidemiology and Health Statistics, School of Public Health, Fujian Medical University, Fuzhou, Fujian 350108, China; Fujian Province Key Laboratory of Environment and Health, School of Public Health, Fujian Medical University, Fuzhou, Fujian 350108, China.

2. Department of Preventive medicine, School of Public Health, Fujian Medical University, Fuzhou, Fujian 350108, China ; Fujian Province Key Laboratory of Environment and Health, School of Public Health, Fujian Medical University, Fuzhou, Fujian 350108, China.

3. Institute of Tropical Medicine, Nagasaki University, Nagasaki 852-8523, Japan

4. Nagasaki Prefectural Institute of Environmental Research and Public Health, Nagasaki 2-1306-11, Japan

5. Department of Public Health, Nagasaki University Graduate School of Biomedical Sciences, Nagasaki 852-8523, Japan

* Corresponding author: Email: hzj99955888@126.com

Supplementary Table 1 100 models selected by R^2^ adjustment：

| Model Selection Criteria Table | | |  |  |  |  |
| --- | --- | --- | --- | --- | --- | --- |
| Dependent Variable: Y | | |  |  |  |  |
| Date: 03/24/17 Time: 09:58 | | |  |  |  |  |
| Sample: 1 439 | |  |  |  |  |  |
| Included observations: 435 | | |  |  |  |  |
|  |  |  |  |  |  |  |
|  |  |  |  |  |  |  |
| Model | LogL | AIC | BIC | HQ | Adj. R-sq* | Specification |
|  |  |  |  |  |  |  |
|  |  |  |  |  |  |  |
| 95 | 594.415349 | -2.709956 | -2.663113 | -2.691467 | 0.962452 | ARDL(1, 1, 0) |
| 100 | 594.393572 | -2.714453 | -2.676979 | -2.699663 | 0.962536 | ARDL(1, 0, 0) |
| 99 | 596.277369 | -2.718517 | -2.671674 | -2.700028 | 0.962772 | ARDL(1, 0, 1) |
| 94 | 599.558692 | -2.729005 | -2.672794 | -2.706820 | 0.963244 | ARDL(1, 1, 1) |
| 90 | 605.439822 | -2.756045 | -2.699833 | -2.733859 | 0.964225 | ARDL(1, 2, 0) |
| 89 | 610.725389 | -2.775749 | -2.710169 | -2.749865 | 0.965002 | ARDL(1, 2, 1) |
| 85 | 611.096074 | -2.777453 | -2.711873 | -2.751570 | 0.965062 | ARDL(1, 3, 0) |
| 98 | 611.075722 | -2.781957 | -2.725746 | -2.759771 | 0.965140 | ARDL(1, 0, 2) |
| 80 | 613.571493 | -2.784237 | -2.709288 | -2.754656 | 0.965376 | ARDL(1, 4, 0) |
| 88 | 613.897247 | -2.785734 | -2.710786 | -2.756153 | 0.965428 | ARDL(1, 2, 2) |
| 93 | 613.747801 | -2.789645 | -2.724065 | -2.763762 | 0.965485 | ARDL(1, 1, 2) |
| 84 | 617.187920 | -2.800864 | -2.725915 | -2.771283 | 0.965947 | ARDL(1, 3, 1) |
| 79 | 620.636415 | -2.812121 | -2.727804 | -2.778843 | 0.966404 | ARDL(1, 4, 1) |
| 83 | 620.639538 | -2.812136 | -2.727818 | -2.778857 | 0.966405 | ARDL(1, 3, 2) |
| 97 | 620.493657 | -2.820660 | -2.755080 | -2.794777 | 0.966539 | ARDL(1, 0, 3) |
| 82 | 622.850757 | -2.817705 | -2.724019 | -2.780728 | 0.966666 | ARDL(1, 3, 3) |
| 87 | 622.806103 | -2.822097 | -2.737780 | -2.788818 | 0.966738 | ARDL(1, 2, 3) |
| 78 | 623.756688 | -2.821870 | -2.728184 | -2.784893 | 0.966805 | ARDL(1, 4, 2) |
| 92 | 622.794042 | -2.826639 | -2.751690 | -2.797058 | 0.966814 | ARDL(1, 1, 3) |
| 96 | 624.300445 | -2.833565 | -2.758616 | -2.803984 | 0.967043 | ARDL(1, 0, 4) |
| 77 | 626.013710 | -2.827649 | -2.724595 | -2.786975 | 0.967070 | ARDL(1, 4, 3) |
| 76 | 626.855060 | -2.826920 | -2.714496 | -2.782548 | 0.967120 | ARDL(1, 4, 4) |
| 81 | 626.799018 | -2.831260 | -2.728205 | -2.790586 | 0.967189 | ARDL(1, 3, 4) |
| 86 | 626.794706 | -2.835838 | -2.742152 | -2.798861 | 0.967265 | ARDL(1, 2, 4) |
| 91 | 626.793970 | -2.840432 | -2.756115 | -2.807153 | 0.967342 | ARDL(1, 1, 4) |
| 75 | 641.225343 | -2.925174 | -2.878331 | -2.906686 | 0.969723 | ARDL(2, 0, 0) |
| 70 | 641.899430 | -2.923676 | -2.867464 | -2.901490 | 0.969746 | ARDL(2, 1, 0) |
| 74 | 644.475276 | -2.935519 | -2.879307 | -2.913333 | 0.970102 | ARDL(2, 0, 1) |
| 50 | 644.725450 | -2.936669 | -2.880457 | -2.914483 | 0.970137 | ARDL(3, 0, 0) |
| 45 | 645.855280 | -2.937266 | -2.871685 | -2.911382 | 0.970222 | ARDL(3, 1, 0) |
| 69 | 646.008290 | -2.937969 | -2.872389 | -2.912086 | 0.970243 | ARDL(2, 1, 1) |
| 25 | 646.968262 | -2.942383 | -2.876803 | -2.916499 | 0.970374 | ARDL(4, 0, 0) |
| 20 | 648.362139 | -2.944194 | -2.869245 | -2.914613 | 0.970494 | ARDL(4, 1, 0) |
| 49 | 648.948854 | -2.951489 | -2.885909 | -2.925605 | 0.970643 | ARDL(3, 0, 1) |
| 44 | 650.301217 | -2.953109 | -2.878160 | -2.923528 | 0.970756 | ARDL(3, 1, 1) |
| 24 | 651.632155 | -2.959228 | -2.884279 | -2.929647 | 0.970935 | ARDL(4, 0, 1) |
| 55 | 652.590988 | -2.959039 | -2.874722 | -2.925760 | 0.970994 | ARDL(2, 4, 0) |
| 65 | 651.695666 | -2.964118 | -2.898538 | -2.938234 | 0.971011 | ARDL(2, 2, 0) |
| 60 | 652.291090 | -2.962258 | -2.887309 | -2.932677 | 0.971023 | ARDL(2, 3, 0) |
| 19 | 652.846267 | -2.960213 | -2.875895 | -2.926934 | 0.971029 | ARDL(4, 1, 1) |
| 30 | 654.958833 | -2.965328 | -2.871642 | -2.928351 | 0.971241 | ARDL(3, 4, 0) |
| 40 | 654.209284 | -2.971077 | -2.896128 | -2.941496 | 0.971277 | ARDL(3, 2, 0) |
| 35 | 654.898734 | -2.969649 | -2.885332 | -2.936370 | 0.971301 | ARDL(3, 3, 0) |
| 5 | 656.142159 | -2.966171 | -2.863116 | -2.925497 | 0.971330 | ARDL(4, 4, 0) |
| 10 | 656.055806 | -2.970372 | -2.876685 | -2.933395 | 0.971386 | ARDL(4, 3, 0) |
| 15 | 655.556688 | -2.972674 | -2.888357 | -2.939396 | 0.971387 | ARDL(4, 2, 0) |
| 64 | 655.959226 | -2.979123 | -2.904174 | -2.949542 | 0.971507 | ARDL(2, 2, 1) |
| 59 | 656.843083 | -2.978589 | -2.894271 | -2.945310 | 0.971556 | ARDL(2, 3, 1) |
| 54 | 657.502425 | -2.977023 | -2.883337 | -2.940046 | 0.971575 | ARDL(2, 4, 1) |
| 39 | 658.739247 | -2.987307 | -2.902989 | -2.954028 | 0.971803 | ARDL(3, 2, 1) |
| 29 | 660.007168 | -2.983941 | -2.880886 | -2.943267 | 0.971835 | ARDL(3, 4, 1) |
| 34 | 659.754554 | -2.987377 | -2.893691 | -2.950401 | 0.971868 | ARDL(3, 3, 1) |
| 14 | 660.110629 | -2.989014 | -2.895328 | -2.952038 | 0.971914 | ARDL(4, 2, 1) |
| 4 | 661.195247 | -2.984806 | -2.872382 | -2.940434 | 0.971922 | ARDL(4, 4, 1) |
| 9 | 660.891688 | -2.988008 | -2.884953 | -2.947334 | 0.971949 | ARDL(4, 3, 1) |
| 51 | 663.559331 | -2.991077 | -2.869285 | -2.943008 | 0.972160 | ARDL(2, 4, 4) |
| 56 | 663.391651 | -2.994904 | -2.882481 | -2.950532 | 0.972204 | ARDL(2, 3, 4) |
| 73 | 660.914983 | -3.006506 | -2.940925 | -2.980622 | 0.972214 | ARDL(2, 0, 2) |
| 52 | 663.557622 | -2.995667 | -2.883244 | -2.951295 | 0.972225 | ARDL(2, 4, 3) |
| 71 | 662.056196 | -3.002557 | -2.918240 | -2.969278 | 0.972230 | ARDL(2, 0, 4) |
| 57 | 663.122485 | -2.998264 | -2.895210 | -2.957590 | 0.972235 | ARDL(2, 3, 3) |
| 63 | 662.102847 | -3.002772 | -2.918454 | -2.969493 | 0.972236 | ARDL(2, 2, 2) |
| 61 | 663.333784 | -2.999236 | -2.896181 | -2.958562 | 0.972262 | ARDL(2, 2, 4) |
| 72 | 661.839351 | -3.006158 | -2.931209 | -2.976577 | 0.972267 | ARDL(2, 0, 3) |
| 53 | 663.433111 | -2.999692 | -2.896638 | -2.959018 | 0.972275 | ARDL(2, 4, 2) |
| 58 | 663.016264 | -3.002374 | -2.908688 | -2.965397 | 0.972287 | ARDL(2, 3, 2) |
| 68 | 662.009388 | -3.006940 | -2.931991 | -2.977359 | 0.972289 | ARDL(2, 1, 2) |
| 62 | 663.043127 | -3.002497 | -2.908811 | -2.965521 | 0.972290 | ARDL(2, 2, 3) |
| 66 | 663.176814 | -3.003112 | -2.909426 | -2.966135 | 0.972307 | ARDL(2, 1, 4) |
| 67 | 662.907334 | -3.006470 | -2.922153 | -2.973192 | 0.972338 | ARDL(2, 1, 3) |
| 26 | 666.464902 | -2.999839 | -2.868678 | -2.948072 | 0.972464 | ARDL(3, 4, 4) |
| 1 | 667.267907 | -2.998933 | -2.858404 | -2.943468 | 0.972500 | ARDL(4, 4, 4) |
| 48 | 663.692631 | -3.014679 | -2.939730 | -2.985098 | 0.972502 | ARDL(3, 0, 2) |
| 31 | 666.264562 | -3.003515 | -2.881723 | -2.955446 | 0.972504 | ARDL(3, 3, 4) |
| 27 | 666.371146 | -3.004005 | -2.882213 | -2.955936 | 0.972517 | ARDL(3, 4, 3) |
| 38 | 664.864794 | -3.010873 | -2.917186 | -2.973896 | 0.972521 | ARDL(3, 2, 2) |
| 28 | 666.022866 | -3.007002 | -2.894578 | -2.962630 | 0.972538 | ARDL(3, 4, 2) |
| 46 | 665.029630 | -3.011630 | -2.917944 | -2.974654 | 0.972542 | ARDL(3, 0, 4) |
| 6 | 667.144538 | -3.002963 | -2.871803 | -2.951196 | 0.972550 | ARDL(4, 3, 4) |
| 2 | 667.243437 | -3.003418 | -2.872258 | -2.951651 | 0.972562 | ARDL(4, 4, 3) |
| 43 | 664.683866 | -3.014638 | -2.930321 | -2.981360 | 0.972563 | ARDL(3, 1, 2) |
| 32 | 666.254249 | -3.008066 | -2.895642 | -2.963694 | 0.972567 | ARDL(3, 3, 3) |
| 36 | 666.254833 | -3.008068 | -2.895645 | -2.963696 | 0.972567 | ARDL(3, 2, 4) |
| 23 | 664.766615 | -3.015019 | -2.930701 | -2.981740 | 0.972574 | ARDL(4, 0, 2) |
| 3 | 666.889409 | -3.006388 | -2.884596 | -2.958319 | 0.972583 | ARDL(4, 4, 2) |
| 13 | 665.907532 | -3.011069 | -2.908014 | -2.970395 | 0.972588 | ARDL(4, 2, 2) |
| 33 | 665.911488 | -3.011087 | -2.908033 | -2.970413 | 0.972589 | ARDL(3, 3, 2) |
| 21 | 665.926205 | -3.011155 | -2.908100 | -2.970481 | 0.972591 | ARDL(4, 0, 4) |
| 41 | 665.989912 | -3.011448 | -2.908393 | -2.970774 | 0.972599 | ARDL(3, 1, 4) |
| 47 | 665.028707 | -3.016224 | -2.931906 | -2.982945 | 0.972607 | ARDL(3, 0, 3) |
| 7 | 667.093195 | -3.007325 | -2.885533 | -2.959256 | 0.972608 | ARDL(4, 3, 3) |
| 11 | 667.143770 | -3.007558 | -2.885766 | -2.959488 | 0.972615 | ARDL(4, 2, 4) |
| 18 | 665.695692 | -3.014693 | -2.921007 | -2.977716 | 0.972626 | ARDL(4, 1, 2) |
| 8 | 666.745588 | -3.010325 | -2.897901 | -2.965953 | 0.972629 | ARDL(4, 3, 2) |
| 37 | 666.243344 | -3.012613 | -2.909558 | -2.971939 | 0.972631 | ARDL(3, 2, 3) |
| 16 | 666.846655 | -3.010789 | -2.898366 | -2.966417 | 0.972642 | ARDL(4, 1, 4) |
| 22 | 665.902591 | -3.015644 | -2.921958 | -2.978668 | 0.972652 | ARDL(4, 0, 3) |
| 42 | 665.981823 | -3.016008 | -2.922322 | -2.979032 | 0.972662 | ARDL(3, 1, 3) |
| 12 | 667.091388 | -3.011914 | -2.899491 | -2.967543 | 0.972673 | ARDL(4, 2, 3) |
| 17 | 666.803158 | -3.015187 | -2.912132 | -2.974513 | 0.972701 | ARDL(4, 1, 3) |
|  |  |  |  |  |  |  |
